# The complete chloroplast genome of *Cymbidium dayanum* (Orchidaceae)

**DOI:** 10.1080/23802359.2021.1934173

**Published:** 2021-06-07

**Authors:** Zhihui Du, Xiyu Yang, Guofei Tan, Zhilin Chen

**Affiliations:** Guizhou Horticulture Institute, Guizhou Academy of Agricultural Sciences, Guiyang, China

**Keywords:** *Cymbidium dayanum*, chloroplast genome, phylogenetic analysis

## Abstract

*Cymbidium dayanum*, a wild orchid species in the Orchid family (Orchidaceae), is considered highly valuable because of its long flowering period and beautiful plant shape. We sequenced the complete chloroplast genome of *C. dayanum* using the Illumina Hiseq platform (Illumina, San Diego, CA). The size of the *C. dayanum* chloroplast genome is 155,408 bp, with an average GC content of 36.76%. This chloroplast genome has containing a large single-copy (LSC) region of 84,189 bp, a small single-copy (SSC) region of 17,991 bp, and two inverted (IRa and IRb) repeat regions of two 26,614 bp. A total of 118 unique genes were annotated, including 76 protein-coding genes, 38 tRNA genes, and 4 rRNA genes. A maximum-likelihood phylogenetic tree indicated that *C. dayanum* is closely related to *C. tracyanum* in the genus *Cymbidium* based on 9 whole chloroplast genome sequences.

*Cymbidium dayanum* is an epiphytic orchid, it mainly grows on trees in open forests or cliffs along streamsides, with altitude ranging between 300 and 1600 m. The flowers have white or cream-yellow sepals and petals, with a maroon stripe in the center (Liu et al. 2006). The flowering period spans for approximately 4 months, from fall to winter, with the inflorescences often blooming asynchronously on each plant and individual flowers commonly lasting for a month (Matsuda and Sugiura 2019). Because of its long flowering period and beautiful plant shape, *C. dayanum* is considered a very valuable wild orchid. *Cymbidium dayanum* has great utilities as a breeding parent of orchids because of its arching and pendulous inflorescence, its utilization as a parent for hybridization of *Cymbidium hybrid*, and successful intergeneric hybridization of *C. dayanum* has been previously recorded (The International Orchid Register: http://apps.rhs.org.uk/horticulturaldatabase/orchidregister). *Cymbidium dayanum* is widely distributed, from Northern India to Japan, South-Eastern Asia and Sumatra (Du and Cribb 2007). In recent years, due to the gradual destruction of ecological environment and excessive collection, the wild population of *C. dayanum* has been drastically reduced (Luo et al. 2008). Therefore, resource conservation and genetic breeding research of *C. dayanum* are in urgent needs, since information about the genome of *C. dayanum* is scarce. In this study, the completed chloroplast genome of *C. dayanum* has been reported for the first time based on illumina pair-end sequencing data, which is useful for genetic utilization of germplasm resources of the family *Cymbidium* in the future.

The fresh samples of *C. dayanum* were collected from Xinyi county of Guangdong province, China(110°57′04″E, 22°21′10″N), and the voucher specimen of *C. dayanum* was stored at the South China Botanical Garden, Chinese Academy of Sciences (Accession no. 14916, e-mail: huangxx@scib.ac.cn). Total genomic DNA was extracted from fresh leaf tissue using the modified CTAB method (Doyle and Doyle 1987), and the isolated DNA was manufactured to average 350 bp paired-end libraries using NEBNext Ultra DNA Library Prep Kit (Illumina, Beijing, China). The constructed libraries were sequenced using NovaSeq platform provided by Benagen Tech Solutions (Wuhan, China), and the collected raw sequences data were quality controlled and removed by the FastQC (Cock et al. 2010). After filtering, approximately 2.2 GB paired-end clean reads were assembled with the program SPAdes (version: 3.13.0) using the *Cymbidium aloifolium* chloroplast genome sequence as reference (Bankevich et al. 2012). Annotations were performed using the online program CPGAVAS2 (Shi et al. 2019). The *C. dayanum* chloroplast genome sequence was submitted to GenBank (Accession no. MW160431).

The complete chloroplast genome sequence of *C. dayanum* was 155,408 bp in length, with a large single-copy (LSC) region of 84,189 bp, a small single-copy (SSC) region of 17,991 bp, and a pair of inverted repeats (IR) regions of 26,614 bp. The overall GC content was 36.76%, whereas the corresponding values of the LSC, SSC, and IR regions were 34.27%, 29.36%, and 43.21%, respectively. A total of 136 gene species were annotated, including 76 protein-coding genes (PCG), 38 transfer RNA (tRNA), and 4 ribosomal RNA (rRNA) gene species. There were 18 genes duplicated in the IR regions, including 6 PCG genes, 8 tRNA genes, and 4 rRNA genes, and the remaining 118 genes were unique genes. Twelve genes contain one intron, while three genes have two introns.

To confirm the phylogenetic status of *C. dayanum*, a molecular phylogenetic tree was constructed using the maximum-likelihood (ML) methods, based on the complete chloroplast genomes of seven *Cymbidium* species and two *Orchidaceae* species as outgroups downloaded from GenBank. ClustalW was applied for multisequence alignment, and the ML analysis was performed using the MEGA6 under the best model of Tamura-Nei model and bootstrapped with 1000 replicates (Vilella et al. 2009). Results showed that ten *Orchidaceae* species were all clustered together (Figure 1). The phylogenetic tree indicated that *C. dayanum* is closely related to *C. tracyanum* supported with 100 bootstrap support values. The characterized chloroplast genome sequence of *C. dayanum* will be helpful for further study on the phylogenetic study, species identification, and genetic engineering.

**Figure 1.**
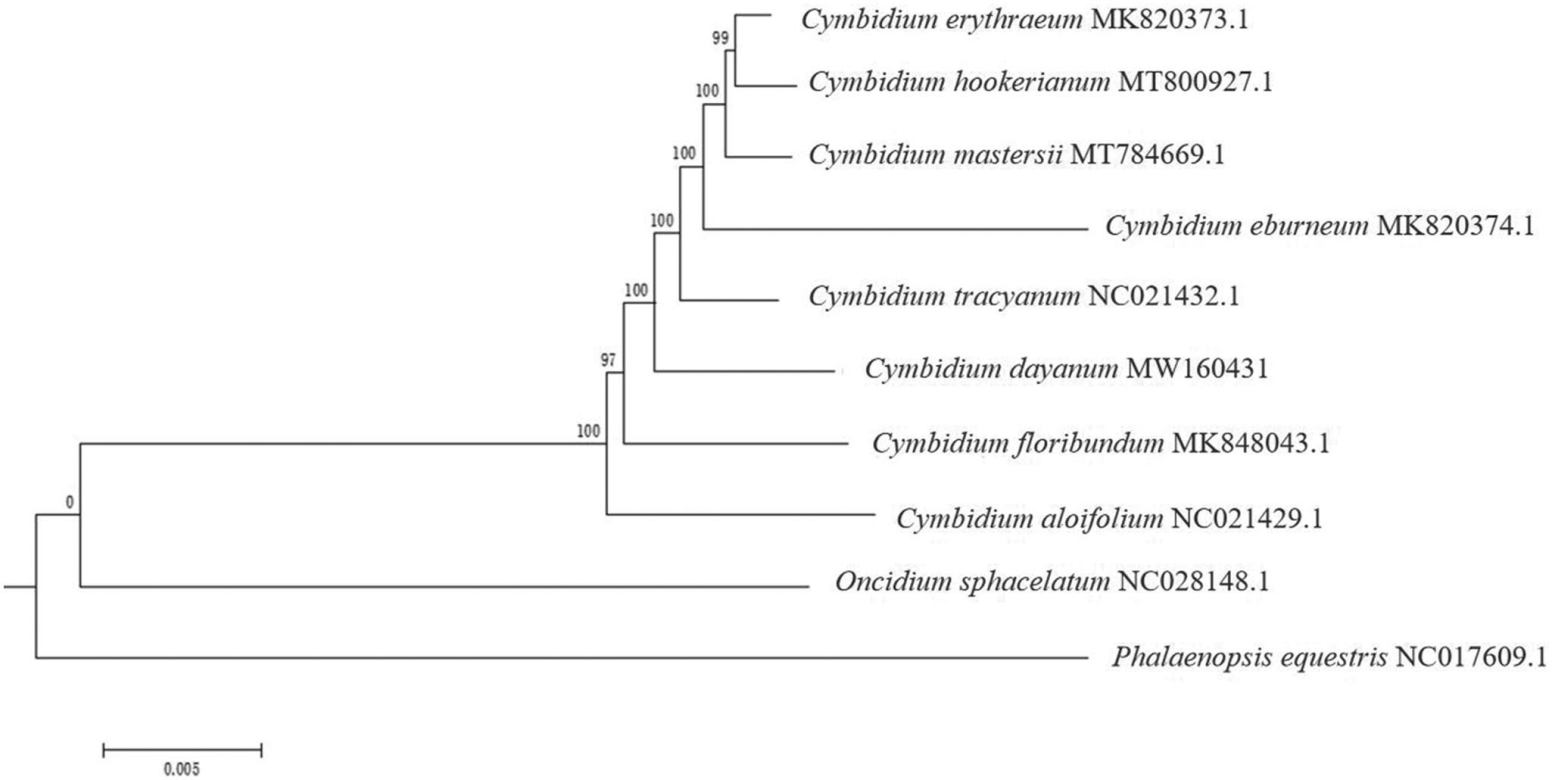
Phylogenetic position of *C. dayanum* inferred by maximum-likelihood (ML) of complete cp genome. The bootstrap values are shown next to the nodes.

## Data Availability

The data that support the findings of this study are openly available in NCBI database at https://www.ncbi.nlm.nih.gov. GenBank Accession no. MW160431, BioProject Accession no. PRJNA718379.
